# Mapping anaerobic sludge bed community adaptations to manure supernatant in biogas reactors

**DOI:** 10.1038/s41598-018-34088-1

**Published:** 2018-10-26

**Authors:** Anna Synnøve Røstad Nordgård, Wenche Hennie Bergland, Rune Bakke, Kjetill Østgaard, Ingrid Bakke

**Affiliations:** 1 Department of Biotechnology and Food Science, Norwegian University of Science and Technology (NTNU), 7491 Trondheim, Norway; 2 Department of Process, Energy and Environmental Technology, University College of Southeast Norway (USN), Kjølnes ring 56, 3918 Porsgrunn, Norway

## Abstract

In upflow anaerobic sludge bed (UASB) reactors, biomass present as granules allows for long solids retention time. Here, granules from a process treating pulp and paper industrial wastewater were successfully applied as inoculum in UASB reactors treating pig manure supernatant, despite high particle content and high ammonium concentrations in the influent. We did a detailed characterization of archaeal and bacterial communities associated with the inoculum and with the aggregated and dispersed fractions of the influent and the reactors after one year of operation. The granular communities underwent major changes and adapted to the highly distinct conditions without disintegration of the granules. Although the granules persisted in the reactors, non-granular aggregates accumulated, and partly replaced the granules. Particles introduced to the reactors by the pig manure influent apparently contributed both as food and biofilm growth support. Archaeal communities in the dispersed reactor phase were similar to those dispersed in the influents, implying successful retention and little loss of archaeal biomass due to detachment or disintegration of granules and other aggregates. Unique bacterial communities developed in the dispersed fraction of the reactors despite of low hydraulic retention times. They probably consisted of fast growing organisms consuming readily degradable organic matter.

## Introduction

Anaerobic digestion (AD) is considered one of the most promising technologies in the field of renewable energy production and has been used to treat organic wastes such as manure for many decades already. Unfortunately, the low energy density of manure gives relatively low production rates in continuous stirred tank reactors (CSTR) currently used for manure AD^1^. Such tanks without efficient biomass retention will be voluminous and expensive to build and operate for a single small-scale farm^1^. Many countries, *e.g*. Germany, transport manure to central AD plants, but this solution is questionable due to the CO_2_ release and cost of transport. Hence, an on-farm solution is needed. We have previously shown that high rate upflow anaerobic sludge bed (UASB) reactors can efficiently treat supernatant from pig manure to obtain sustainable energy recovery^2^ despite the solids content being well above the levels considered appropriate as UASB reactor influent^2,3^. Granules are one of the main components of such high rate AD. Generally, microorganisms including bacterial acidogens and acetogens and archaeal methanogens, aggregate into granules of 0.5–3 mm size, giving a sedimentation velocity high enough to avoid washout even under high hydraulic load^4^. In our UASB reactors treating manure supernatant, granules originating from a UASB reactor treating wastewater with high carbon to nitrogen (C/N) ratio from the pulp and paper industry were successfully applied as inoculum. Suspended solids accumulated in the reactors and formed a solid fraction together with the granules^2^. It is not yet clear to what extent the accumulating solids played a role in methanogenesis, and authorities on UASB even claim that suspended solids rich feeds should be avoided in granular sludge bed processes for wastewater treatment^3^. Given this, there is very little directly relevant literature on the topic investigated here, while there are large quantities of slurries that can be used for biogas production. This study is therefore part of an effort to vastly expand the applicability of high rate sludge bed AD.

High ammonia concentrations are well known to inhibit methanogenesis. Compared to the hydrogenotrophic methanogens, the aceticlastic methanogens (*Methanosaeta* and *Methanosarcina*) are considered sensitive to high ammonia levels. At high ammonia concentrations, syntrophic acetate oxidizing bacteria (SAOB), and subsequent methane production by hydrogenotrophic methanogens, has been suggested to be the major mechanism for acetate conversion in AD. We recently characterized archaeal and bacterial communities in UASB reactors treating pig manure supernatant during adaptation to high ammonia concentrations^5^. *Methanosaeta* was found to correlate with methanogenesis at high ammonia concentrations. This was surprising, since *Methaonsaeta* has previously been found to be more sensitive to high ammonia concentrations than *Methanosarcina*^6–8^. Moreover, a monophyletic group of OTUs, related to Thaumarchaeota, was highly abundant at high ammonia concentrations. In the present study, the objective was to characterize microbial communities in the granular and non-granular aggregates and dispersed communities in the liquid phase in these UASB reactors after one-year adaptation to treatment of pig manure supernatant at high ammonium concentrations.

We aimed at answering the following questions: 1) How did the granular communities adapt to the particle rich pig manure supernatant used as influent in the reactors and to ammonia enrichment? 2) Did the accumulating solids in the various phases in the reactors establish distinct methanogenic communities? 3) Considering the low hydraulic retention time (HRT), did unique dispersed communities develop in the reactors’ liquid phase? Illumina sequencing of 16S rRNA gene amplicons was applied for detailed characterization of bacterial and archaeal communities associated both with liquid fraction and the aggregates in the solid fraction of both the influents and reactors.

## Materials and Methods

### Reactor influent, inoculum and operation

The manure substrate was collected from a pig production farm in Porsgrunn, Norway, and handled as described by Bergland, *et al*.^2^. After collection, the manure substrate was stored at 4 °C until use. Four laboratory scale reactors (denoted High Ammonia (HA1 and HA2), and Low Ammonia (LA1 and LA2)) were fed pig manure slurry supernatant (hereafter referred to as pig manure) for 358 days. Set up and operation has been described previously^5^. The reactors were operated at hydraulic retention time (HRT) 1.0 day. The organic loading rate (OLR) was 16 ± 2 g COD L^−1^ d^−1^ for the entire experiment. Urea was added to the substrate for two of the reactors (HA1 and HA2) from day 69 to increase the concentration of total ammonia nitrogen (TAN, *i.e*. NH_4_^+^  + NH_3_) and resulted in 3.7 ± 0.2 g NH_4_^+^ − N L^−1^. Two reactors (LA1 and LA2) were fed pig manure supernatant as collected, resulting in TAN concentrations of 1.9 ± 0.2 g L^−1^. Operational variables are summarized in Table 1. Measurements of chemical oxygen demand (COD), pH, volatile fatty acids (VFA), NH_4_^+^ − N, gas composition and methane production were performed as described by Bergland *et al*.^2^.

**Table 1:** Properties of the influent and reactors given as average ± standard deviation (SD).

Property	HA reactors	LA reactors
	average ± SD	average ± SD
pH of the influent	8.7 ± 0.1	7.6 ± 0.2
NH_4_ − N in influent	3.7 ± 0.2 g L^−1^	1.9 ± 0.2 g L^−1^
TAN in the reactors	3735 mg L^−1^	1840 mg L^−1^
FAN in the reactors	903 mg L^−1^	176 mg L^−1^
Acetate influent-effluent	3.2-2.1 g L^−1^	3.2-0.3 g L^−1^
VFA influent	5.5 ± 1.9 g L^−1^	5.5 ± 1.9 g L^−1^
VFA removal	51%	92%
COD_T_ influent	21 ± 4.8 g L^−1^	21 ± 4.8 g L^−1^
COD_T_ removal	49%	58%
Methane yield	1.65 NL CH_4_ L^−1^ influent	3.00 NL CH_4_ L^−1^ influent

Total ammonia nitrogen (TAN, *i.e*. NH_4_^+^ + NH_3_), free ammonia nitrogen (FAN, *i.e*. NH_3_), acetate, COD_T_ and methane yield are given for experimental day 347.

FAN was calculated:

$$FAN=\frac{TAN}{1+\frac{{10}^{-pH}}{{K}_{a}}}\,\,\,\,{\rm{where}}\,{K}_{a}=\frac{[N{H}_{3}][{H}^{+}]}{[N{H}_{4}^{+}]}$$

*FAN* is the concentration of free ammonia nitrogen, and *TAN* is the concentration of the total ammonia nitrogen.

The granules used as inoculum originated from a UASB reactor treating pulp and paper process wastewater at Norske Skog Saugbrugs in Halden, Norway, characterized by HRT less than 0.5 days, low levels particulate matter, OLR 10–20 g COD L^−1^ d^−1^, and temperature 35 °C. Ammonia and other nutrients are added to avoid nitrogen deficiency at the Saugbrugs wastewater treatment process, maintaining ammonia <10 mg/L to comply with discharge limits. The granules used as inoculum had a diameter of 1–2 mm and filled 2/3 of reactor volumes.

### Sampling and DNA extraction

Three samples of granular sludge from the pulp and paper industry, representing differential storage periods after collection at 11 °C, were analyzed; the first sampling was performed immediately upon arrival from the pulp and paper factory, and the second and third sampling after 6 and 12 months of storage, respectively. The reason for including these samples were twofold: First, to see whether the granular communities changed during storage. Second, this allows for assessing the variability in the granular communities among samples (as opposed to compare the microbial communities of the adapted granules to only one inoculum sample). The third sampling represented the inoculum of the reactors. All three samples are referred to as the pulp and paper (PP) granules.

Samples were collected from both the HA and LA influents and all four reactors on experimental day 341 (D341) and 347 (D347) of the 358 days long experiment as described by Nordgård *et al*.^5^. To be able to analyze microbial communities associated with different fractions of the samples, the samples were processed into aggregated particulate matter, granules and dispersed phases as follows: For the aggregated particulate phase, samples collected on D341 and D347 were centrifuged at 200 g for 10 minutes. The pellets were resuspended in 1x phosphate buffer saline (PBS). These steps were repeated twice before a final centrifugation at 4000 g for 10 minutes to remove liquid. The resulting pellet represents the aggregated particulate phase.

For the liquid phase, samples collected at D347 were added PBS (1x) up to 50 ml and centrifuged at 200 g for 10 minutes. The pellets were discarded, the liquid volume adjusted with PBS, and centrifuged again at the same conditions. The supernatant, representing the liquid phase, was transferred to clean tubes and centrifuged at 12 000 g for 20 minutes. The pellets represented the microbes dispersed in the liquid phase. To sample granules from the reactors (hereafter referred to as reactor granules (Rgr)), they were picked by forceps from HA1 and LA1 reactor samples (D341) and rinsed with PBS (1x).

Total DNA was extracted from all samples directly after this processing, using the Power Soil DNA isolation kit (Mobio Laboratories Inc., Carlsbad, CA, USA) as described by the manufacturer.

### PCR and DNA sequencing

The v3 and v4 region of both the bacterial and archaeal 16S rRNA gene was amplified by PCR, and amplicons were generated and indexed for all samples as described by Nprdgård *et al*.^5^. The amplicons analyzed in this study were part of two larger amplicon libraries representing bacterial and archaeal communities, each consisting of a total of 23 amplicons, respectively, and were sequenced on one lane on an Illumina MiSeq Instrument. The sequencing data were processed with the high performance USEARCH utility. The processing included demultiplexing, quality trimming (Fastq_filter command with an expected error threshold of 1), and chimera removal and clustering at the 97% similarity level by the UPARSE-OTU algorithm. Taxonomy assignment was based on the Utax script with a confidence value threshold of 0.8 and the RDP reference data set (version 15). Two operational taxonomic unit (OTU) tables (Archaea and Bacteria) were generated as described previously^5^. The resulting Illumina sequencing data are available at the European Nucleotide Archive (accession numbers ERS1982799-ERS1982809, ERS1982775-ERS1982786, ERS1982728-ERS1982738, and ERS1982704-ERS1982715).

### Statistical analyses

All statistical analyses were performed using the program package PAST version 2.17^9^ as described in Nordgård *et al*.^5^. Community structure was compared between samples by calculating Bray-Curtis similarities^10^. Principal coordinate analysis (PcoA) was based on ordinations of Bray-Curtis similarities. PERMANOVA was used for testing differences in average Bray–Curtis dissimilarities between groups of samples^11^. SIMPER (Similarity Percentage) analysis was employed to identify taxa primarily responsible for differences between two or more sample groups^11^.

### Ethical statement

Compliance with Ethical Standards. Anna Synnøve Røstad Nordgård declares that she has no conflict of interest. Wenche Hennie Bergland declares that she has no conflict of interest. Rune Bakke declares that he has no conflict of interest. Kjetill Østgaard declares that he has no conflict of interest. Ingrid Bakke declares that she has no conflict of interest. This article does not contain any studies with human participants or animals performed by any of the authors.

## Results

All reactors had constant high organic loading rate and produced methane with higher methane yields in LA than HA reactors^5^. The HA reactors were initially strongly inhibited by the high ammonia concentrations (<10% of the yield in LA). However, a substantial increase in the methane yield for the HA reactors was observed after around 300 days of adaptation, after which the final samples of the present study were collected. TAN and FAN concentrations, as well as CODT removal and methane yield, is given for experimental day 347 in Table 1. Details about the reactor performance throughout the experimental period are given in Nordgård *et al*.^5^.

### Sequencing effort and microbial diversity

**Figure 1:**
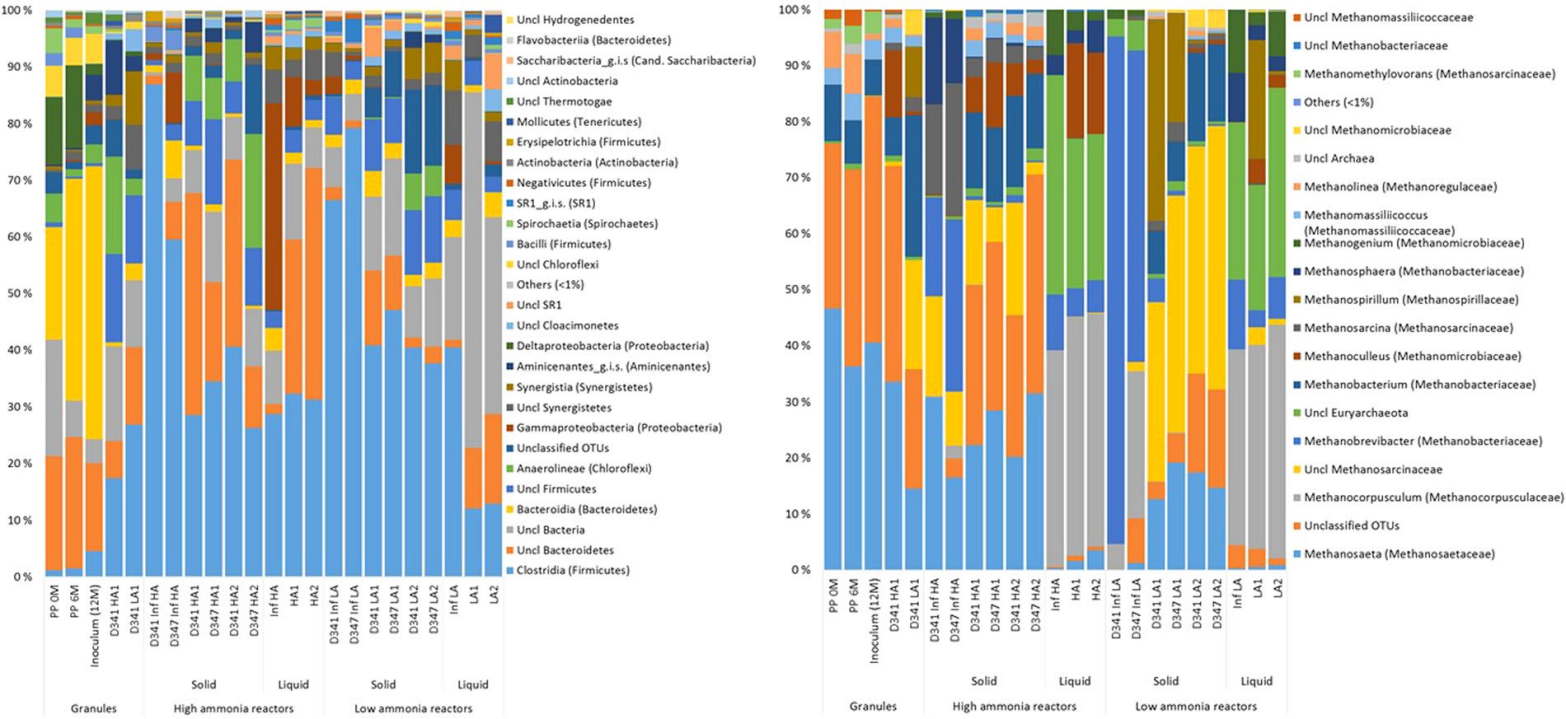
Relative abundances of bacterial classes (**A**) and archaeal genera (**B**) represented in the v3-4 16S rRNA gene amplicons obtained for individual influent, reactor, and granular samples. Each bar represents one sample. D: day; PP: pulp and paper granules; Inf HA: influent to HA reactors; Inf LA: influent to LA reactors; *Cand*.: *Candidatus*; *g.i.s*.: *genera_incertae_sedis*. “Unclassified” indicates that OTUs could not be classified at the domain level, “Uncl” indicates that OTUs could not be classified at class level (**A**) or family level (**B**). Only taxa represented by a portion of ≥1% of the sequence reads in at least one of the samples are shown. “Others” includes all reads representing the taxa with lower abundance in all samples.

The OTU tables yielded 2446 and 121 OTUs assigned to *Bacteria* and *Archaea*, respectively. The number of reads for each sample is given in Table S1. Estimated (Chao1) and observed OTU richness are compared and indicate a coverage of 73.2% and 92.4% on average of the estimated bacterial and archaeal richness, respectively (Fig. S1). Richness (Chao1), evenness, and Shannon’s diversity suggest a considerable increase of diversity for granular bacterial communities after nearly one year in the reactors. A similar trend, although less pronounced, is found for the archaeal communities. Generally, the richness of the bacterial communities were around 10-fold higher than for the archaeal communities (Fig. S1). A summary of the communities at the class level shows that *Clostridia* (*Firmicutes*), *Bacteriodetes* (OTUs classified at phylum level) and OTUs classified only at the domain level (*Bacteria*), were generally the three most abundant taxa for the reactor samples (Fig. 1A). The abundance of archaeal genera varied among samples, but Methanosaeta was generally abundant in most aggregate samples, while *Methanocorpusculum* was highly abundant among the dispersed archaea. Furthermore, a strikingly high fraction of the archaeal OTUs, particularly for the granule and total aggregate HA samples that had been exposed to extreme ammonia concentrations, could not be taxonomically assigned below the domain level (Fig. 1B).

### Granular microbial communities

The granules used as inoculum originated from a pulp and paper industrial process and were subjected to fundamentally different selection regimes in the process used here, characterized by higher ammonia concentrations and higher content of particulate solids. These changes led to a shift from a purely granular sludge culture to a new stable situation where only about half the reactor microbial aggregates consisted of granules. At the start of the experiment, the granules filled around 2/3 of the reactor volumes. The sludge bed hight remained quite stable (2/3 of reactor volumes), but the granules were partly replaced by particles from the feed, constituting about 1/2 of the sludge bed biomass. The remaining granules maintained shape and size (1–2 mm). A key question was therefore, given that the granules survived these rater extreme changes even at the highest ammonia levels, how did the communities within the granules adapt to these conditions?

**Figure 2:**
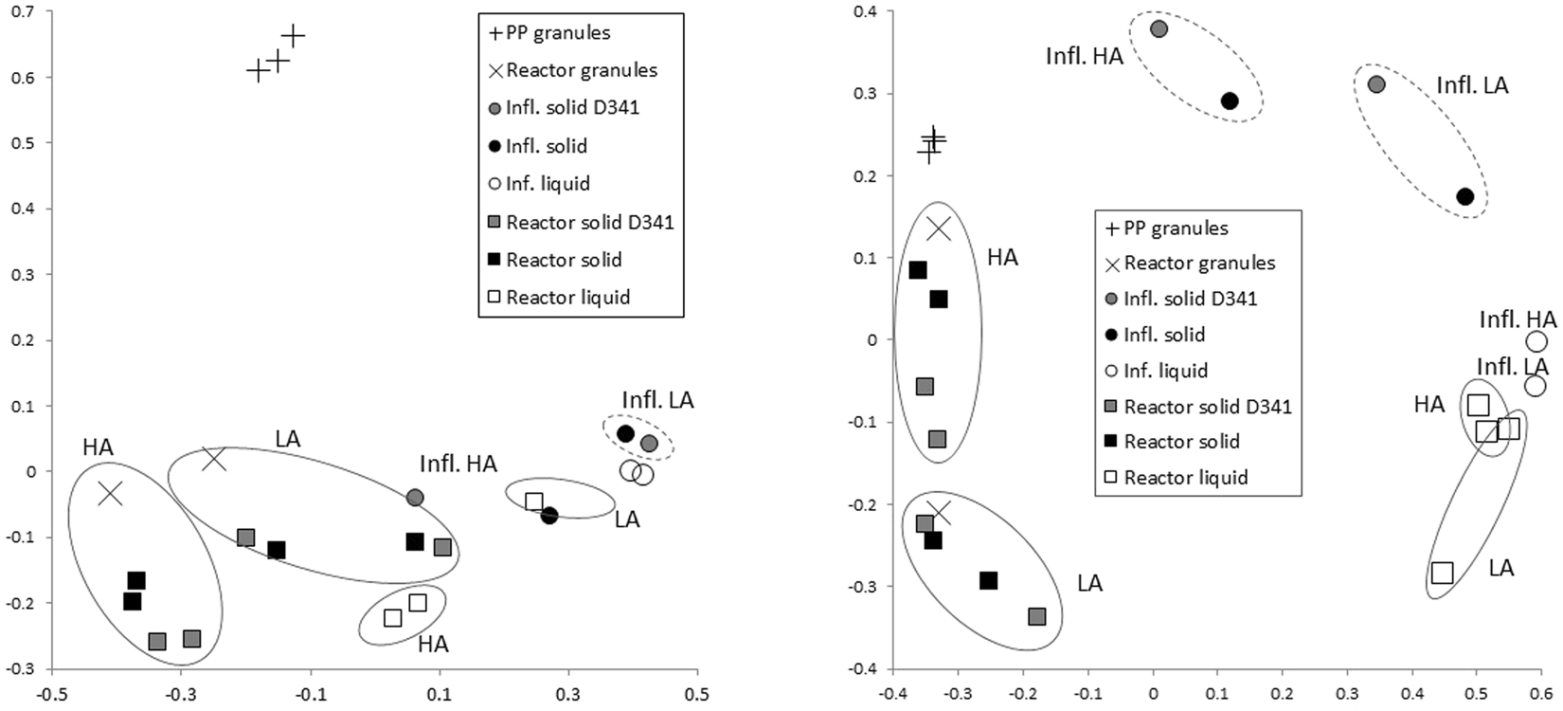
Principal coordinate analysis ordination based on Bray-Curtis similarities for (**A**) bacterial and (**B**) archaeal community profiles associated with granule samples and for liquid and solid fractions of influent and reactor samples. PP granules were sampled 12, 6, and 0 months prior to the experiment. Reactor granules were sampled on experimental day 341 (D341). All other samples were taken at day 347. Solid line circle indicate samples from the reactors while dashed line circles indicate samples from the influents.

**Figure 3:**
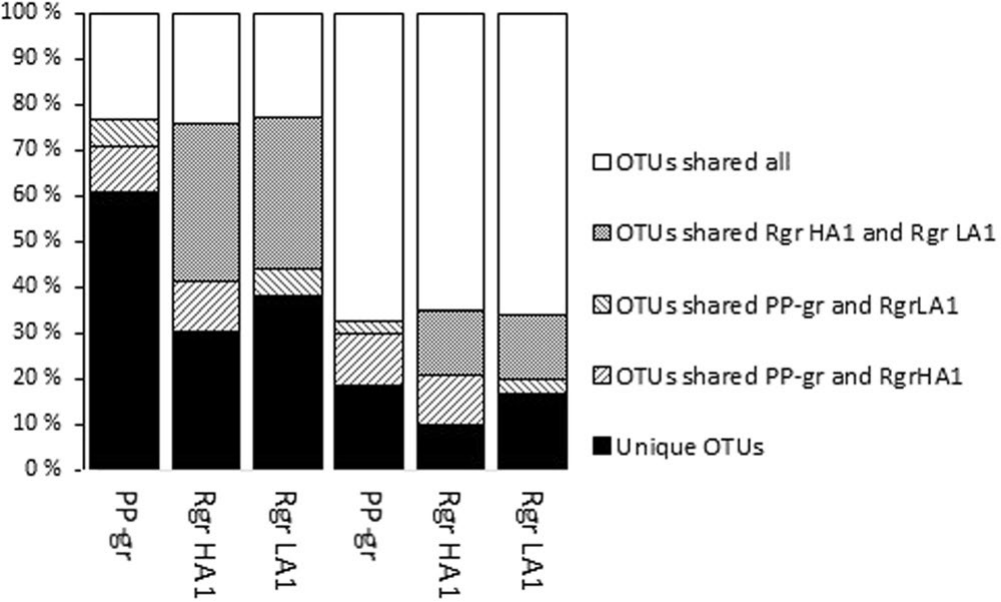
The percentage of unique and shared OTUs between the pulp and paper granules (PP gr) and reactor granules (Rgr) in samples from reactor LA1 and HA1.

Principal coordinate Analysis (PcoA)(Fig. 2) illustrated that the bacterial community profiles in the PP granules were clearly distinct from those of the reactor granules after one year of operation. Moreover, very low Bray-Curtis similarities indicated major changes of the bacterial communities (0.10 ± 0.05 and 0.07 ± 0.02 for PP granules versus the HA1 and LA1 granules, respectively; Table 2). It was also interesting to note that only a small fraction of the bacterial OTUs were shared by the PP and reactor granules (around 5–10%; Fig. 3), and as much as 60% of the bacterial PP granular OTUs were unique to the PP granules (Fig. 3). A marked change in the community composition was a substantial decrease in the relative abundance of *Bacteroidia* and *Deltaproteobacteria*, from an average of 35 ± 14% and 9 ± 7% in the PP-granules to an average of 1.8 ± 1.6% and 0.5 ± 0.4% in the reactor granules, respectively. The abundance of *Clostridia* increased dramatically from 2.4 ± 1.8% to 21 ± 7% on average, thereby being the most abundant bacterial class in the reactor granules (Fig. 1A). At the OTU level, SIMPER analysis showed that an OTU classified as *Bacteroidales* contributed most to the Bray-Curtis dissimilarity between the PP and the reactor granular communities (Table S2). Its abundance was strongly reduced (mean abundance 34.7% in PP granules) and accounted for only 0.05% (HA1) and 0.02% (LA1) of reads in the reactor granular communities (Table S2). Furthermore, abundances of an *Anaerolineae* (*Chloroflexi*) OTU and an OTU classified only as *Bacteria* were strongly increased in the HA1 reactor granules, while abundances of *Synergistetes, Clostridiales*, and *Bacteroidetes* OTUs showed the largest increase in the LA1 reactor granules (Table S2).

**Table 2:** Average Bray-Curtis similarities with standard deviation for comparisons of community profiles between samples.

	Bacteria	Archaea
	Average ± SD	Average ± SD
*Granules*		
PP granules vs Reactor granules HA1	0.10 ± 0.05	0.70 ± 0.01
PP granules vs Reactor granules LA1	0.07 ± 0.02	0.39 ± 0.02
Reactor granules HA1 vs Reactor granules LA1*	0.31	0.46
Solid D341 HA1 vs Reactor granules HA1*	0.36	0.68
Solid D341 LA1 vs Reactor granules LA1*	0.20	0.54
*Reactor and influent*		
HA reactor liquid vs Influent HA liquid	0.30 ± 0.05	0.72 ± 0.13
LA reactor liquid vs Influent LA liquid	0.27 ± 0.09	0.73 ± 0.01
HA reactor solid vs Influent HA solid	0.21 ± 0.07	0.27 ± 0.04
LA reactor solid vs Influent LA solid	0.14 ± 0.05	0.04 ± 0.03
Influent samples only*		
Solid D347 HA vs Liquid HA	0.27	0.18
Solid D347 LA vs Liquid LA	0.26	0.49
*Reactor samples only*		
Solid D347 HA vs Liquid HA	0.28 ± 0.04	0.11 ± 0.05
Solid D347 LA vs Liquid LA	0.26 ± 0.09	0.12 ± 0.11

HA: High ammonia reactors; LA: Low ammonia reactors; PP: pulp and paper; D341 and D347: day 341 and 347 of the experiment. Samples for which experimental day is not given were all sampled at day 347.

*Only one comparison, therefore no SD.

The archaeal communities associated with the granules also changed, but less profoundly. Nearly 70% of the archaeal OTUs were common to all the granular samples (Fig. 3). Surprisingly, PcoA ordination indicates that archaeal communities were relatively similar between the HA1 reactor (granules) and the PP granules, even though these were exposed to the largest difference in ammonia. The archaeal communities differed more between the HA1 and the LA1 reactor granules. This was corroborated by Bray-Curtis similarities; which were found to be 0.70 ± 0.01 and 0.39 ± 0.02 for comparisons between HA1 and LA1 to the PP granules, respectively (Table 2). Moreover, Bray-Curtis similarities were relatively low for comparisons between the HA1 and LA1 reactor granules (0.46). The distinct ammonia concentrations in the HA and LA reactors is probably the reason for the different adaptation of the archaeal granular communities between these reactors. The archaeal communities in the PP and HA1 granules were dominated by the aceticlastic genus *Methanosaeta* and OTUs that could not be classified at domain level, combined accounting for more than 70% of the reads on average (Fig. 1B). For the LA1 granules, *Methanosaeta* abundance decreased notably (from 40.5% in the inoculum to 14.4%), while abundances of *Methanosarcinaceae*, *Methanobacterium*, and *Methanospirllium* increased (Fig. 1B). According to SIMPER analysis, the most important changes of the granular archaeal communities at the OTU level was a strong increase in the abundance of a *Methanoculleus* OTU in HA1 granules (from 0.002 to 11.9%; Table S3), and reduction of a *Methanosaeta* OTU in the LA1 granules (from 40.5–11.44%; Table S3). Abundances of OTUs representing *Methanosarcinaceae* and *Methanobacterium* increased strongly in the LA1 granules (from 0.003 to 19.3% and from 5.7 to 25%, respectively; Table S3).

### Microbial communities in the aggregated biomass phase

Visual inspection of the reactor sludge beds revealed that non-granular particles made up around half of the aggregated sludge bed at end of the experiment. Evidently, the influent manure slurry supplied organic particles, probably serving both as feed and substratum for biofilm growth. The communities associated with the total sludge bed aggregated biomass were characterized to learn more about the colonization and degradation of the slowly degradable biofilm carriers supplied by the feed.

Low Bray-Curtis similarities suggest highly dissimilar microbial communities associated with the aggregated fractions of the influent and the reactors for *Archaea* and *Bacteria* (Table 2). Thus, the accumulating aggregates in the reactor did not represent the microbes associated with particle-rich manure supernatant influent. We further compared the microbial communities in the reactor granules to those of the total aggregated reactor phase. The archaeal communities were relatively similar, as illustrated by the PcoA plot (Fig. 2B) and Bray-Curtis similarities (Table 2). Methanogenic genera, such as *Methanosaeta* and *Methanobacterium* were generally abundant in both total and granular aggregated fractions (Fig. 1B). *Methanospirillum* and *Methanosarcinaceae* (not classified at the genus level) were abundant in both the total aggregated and granular fractions of LA reactors, and *Methanoculleus* were abundant in both samples types in the HA reactors. The high abundance of these taxa in total and granular aggregates implies active methanogenic communities in addition to the granules. Thus, it appears that the methanogenic communities in the biofilms developing on particles from the influent had more similarities with the granules than with the influent communities. Bacterial communities, however, differed more between the granules and the total aggregate fractions in the reactors (Figs 1B, 3A), with relatively low Bray-Curtis similarities (0.36 for HA1 and 0.2 for LA1; Table 2).

### Dispersed microbial communities

The HRT of this process was as low as one day, which is less than the generation time expected for many of the microorganisms involved in AD. We therefore asked whether unique dispersed microbial communities developed in the reactors, or whether they simply reflected the microbial communities introduced by the influent or the microbial communities of the aggregates due to detachment of microorganisms from these.

The dispersed archaeal communities of the reactor samples were found to be strikingly similar to those of found dispersed in the influent samples as illustrated by PcoA plot (Fig. 2B) and high Bray-Curtis similarities (on average over 0.70; Table 2). Particularly *Methanocorpusculum* and OTUs classified only at phylum level (*Euryarchaeota*) were abundant in both influent and reactor samples (Fig. 2). Hence, specific dispersed archaeal communities did not develop in the reactors. Furthermore, the dispersed archaeal communities were distinct from those of the particulate fraction, and thus obviously not much influenced by detachment from the microbial aggregates in the sludge beds nor in the influent.

The situation was different for the bacterial communities. PcoA analysis indicated that the dispersed bacterial communities in the reactors and influents differed considerably (Fig. 2A), and Bray-Curtis similarities for comparisons between these samples were low (around 0.3, Table 2). According to SIMPER analysis, an OTU classified only at the domain level, contributed most (15%) to the Bray-Curtis dissimilarity between dispersed communities in LA reactors and the corresponding influent. This OTU amounted to 31.1% on average in the dispersed LA reactor communities, but was not detected in the influent samples. Both Utax and RDP Classifier suggested that this OTU represented *Clostridiales*, but at low confidence thresholds. Another OTU, classified only at the phylum level as Bacteroidetes explaining 14% of the dissimilarity between the dispersed communities of the HA reactor and influent, accounted for 30.5% of the reads for the dispersed HA reactor samples, but was not observed in influent samples. As the dispersed bacterial communities in the reactors also were highly dissimilar from the aggregated ones, unique dispersed bacterial communities apparently developed in the reactors.

## Discussion

Granules are essential for the process efficiency in UASB reactors, and it is generally assumed that particle rich feeds are not suitable for UASB reactors^12^. Bergland, *et al*.^2^ demonstrated however, that UASB reactors could handle particle rich feeds. Here we investigated the adaptation of granular communities in response to particle rich pig manure supernatant used as feed. The pulp and paper industrial process, from which the granules originated, was characterized by low ammonium concentration and low levels of particles, while here, the granules were subjected to much higher content of solids and elevated ammonia concentrations. Manual inspection of the reactor sludge showed that the granules persisted in the sludge bed of the reactors after around one year of operation, but that they were partly replaced by particles. The amplicon sequencing analysis illustrated that particularly the bacterial communities of the granules underwent major changes in the reactors. Apparently, this did not result in a disintegration of the granular structure, and the microbial communities that developed were compatible with maintenance of the granular structure. The reactors were operated for still one more year after the last sampling time reported here, and we observed that the granules remained as a stable part of the reactors’ sludge beds, accounting for around half of the total solids (data not shown). Other studies have reported major changes in granular species inventory due to other kinds of adaptation^13,14^, but they did not comment on granule abundance in the reactors after completing the adaptation. Another surprising observation is that the methanogenic community that finally handled extreme ammonia levels after a year of adaptation (HA1) was more similar to the inoculum community from a process with very low ammonia (PP) than with the culture that adapted to an ammonia level that can cause moderate inhibition (LA1). The main reason for this similarity is a marked increase in the *Methanosarcinaceae* and a decrease in the *Methanosaeta* abundance in the LA granules, while the abundance of the aceticlastic *Methanosaeta* remained relatively high in the HA granules. This appears to contradict previous studies, which reported decreasing abundances of the obligate aceticlastic *Methanosaeta* with increasing concentrations of ammonia^15–17^. Calli, *et al*.^18^ suggests that loss of *Methanosaeta* activity at high ammonia levels is due to loss of filamentous growth. The filamentous *Methanosaeta* has been suggested to have an important role in the formation and maintenance of stable anaerobic granules^19^. A possible explanation for the apparent high ammonia tolerance for *Methanosaeta* in the HA reactors, could be protection obtained by growing in aggregates with other microbes in the sludge granules. The higher abundance of *Methanosarcina* in LA, on the other hand, could be due to competitive advantages of *Methanosarcina* compared to *Methanosaeta*. The FAN concentration in LA reactors was 0.14 ± 0.10 g NH_3_-N L^−1^, which is below the ammonia threshold of 600 mg L^−1^ for which *Methanosarcina* cell clusters are expected to disintergrate^6^. *Methanosarcina* also has a higher growth rate than *Methanosaeta* (0.60 versus 0.20 d^−1^, respectively)^6,20^, more extensively discussed by Nordgård *et al*.^5^. We also observed an increase in *Methanoculleus* in the HA granules compared to the PP granules. Increases in these genera during high ammonia concentrations is in accordance with literature^16,21^.

The abundance of *Clostridia* increased markedly in the reactor granules. The Clostridiales has previously been recognized as one of the most abundant bacterial orders in AD, and members of this order are involved in hydrolysis, acidogeneisism and acetogenesis steps (see De Vrieze and references therein^22^). The class Clostridia was apparently selected for in the UASB reactors. Clostridia has previously found to dominate the bacterial communities in thermophilic AD^23,24^. In a study of 29 different full-scale anaerobic digestion installations, De Vrieze *et al*.^22^ also found that high Clostridia abundances were related to high concentrations of FAN. In line with these findings, Li *et al*.^25^ found a positive correlation between high FAN concentrations and the abundance of Clostridium. Compared to the bacterial community composition in the PP granules, the abundance of Bacteroidia decreased in the granules during the year of operation. This class has previously been found have similar functions as Clostridia in AD (e.g. butyrate fermentation, propionate and xylose degradation, and glycolysis; see Joyce *et al*.^26^). A possible interpretation of these observations is that members of Clostridia take over functions previously performed by Bacteroidia (in the PP granules) in our reactors. Another striking observation was the large fraction of reads that could not be classified below domain level. As discussed in Nordgård *et al*.^5^, we found indications that one of the most abundant unclassified OTU might be related to Hydrogenedentes, and others to Clostridiales and Chloroflexi. This suggests that we still have limited knowledge about important bacterial players in AD.

The non-granular solid fraction is especially interesting in this process, since the feed used here (settled pig manure) has a particle content much higher than recommended for UASB treatment^12^. The accumulation of non-granular solids in the reactors during the experiment might indicate a potential function in the AD process. The communities associated with the aggregated reactor fractions resembled those of the granular communities, particularly for *Archaea* (Table 2, Fig. 2B). Similar abundances of *Methanosaeta* were found in the reactor granules and the total aggregated fraction of the reactors (Fig. 1B). This strongly supports the conclusion that archaeal communities present in the accumulating aggregated fraction did contribute to methane production. Bergland *et al*.^2^ observed that a significant fraction of such influent particles were converted to methane under similar conditions as those tested here. The influent particles probably served as support for biofilm growth, and a significant fraction of these particles were organic matter degraded in the AD process^2^.

Dispersed archaeal communities were similar in the reactor and influent liquid fractions, indicating that distinct dispersed archaeal communities did not develop in the reactors. Due to the slow growth of methanogenic archaea, they would not be expected to able to grow planktonically at the low HRT (1 d) applied. This also implies that the reactor effluents, typically dominated by the dispersed organisms, were not strongly influenced by detachment from or disintegration of granules and non-granular aggregates.

Distinct bacterial communities developed, on the other hand, in the dispersed reactor fractions despite low HRT; shaped by the selection pressure and conditions in the reactors. These communities (Fig. 1A) probably utilized easily degradable organic matter in the dispersed phase, and thereby contributed to the AD process. The LA reactors especially were dominated by OTUs that could only be classified on higher levels and that were not detected in the influents (Fig. 1A). High abundances of unclassified OTUs indicate that much remains to be uncovered about microbial interactions and taxonomy in anaerobic digestion.

In summary, the granular archaeal and bacterial communities showed high abilities to adapt to changes in selection regimes and to be functional under conditions very different from those in the process from which they originated. The abundance of the reactor granules decreased, while a non-granular aggregated fraction accumulated in the reactors, to constitute about half of the retained sludge bed biomass. Archaeal communities showed high degrees of similarity between the granules and the total solid fractions of the reactors. We propose that the particles introduced to the reactors by the pig manure influent contributed in the AD process by providing support for biofilm growth, thereby contributing to digestion of both suspended and dissolved organics. These biofilm aggregates replace a large fraction of the granules in the sludge beds but not more than about half, establishing a balance between the two types of aggregates, probably with significant interaction, as reflected by community similarities. Interestingly, despite the low HRT, the bacterial communities of the liquid reactor fraction differed considerably from those of the influent. Bacteria dispersed in the reactor liquids had little resemblance with the other fractions, probably representing a fast growing population consuming readily degradable organic matter.

## Electronic supplementary material

Supporting information

## Data Availability

The datasets generated during and analysed during the current study are available from the corresponding author on reasonable request.
