# Supplementary material for: Mapping anaerobic sludge bed community adaptations to manure supernatant in biogas reactors
Source: Sci Rep. 2018 Oct 26;8:15870. doi: 10.1038/s41598-018-34088-1 (PMC6203818; doi:10.1038/s41598-018-34088-1)
Supplement: Supplementary file 1 — Supporting information [file 41598_2018_34088_MOESM1_ESM.docx]

**Supporting information to**

**Mapping anaerobic sludge bed community adaptations to manure supernatant in biogas reactors**

Anna Synnøve Røstad Nordgård1, Wenche Hennie Bergland2, Rune Bakke2, Kjetill Østgaard1 and 4 Ingrid Bakke1

**Supporting information**

**Table S1.** Number of reads per sample after quality filtering and chimera removal, obtained with the primers B-338F/B-805R amplifying bacterial DNA and A-340F/A-760R amplifying archaeal DNA. The pulp and paper granules (PP gr.) were sampled upon arrival and after 6 and 12 months in storage at 11 °C. The reactor granules were sampled from the HA1 and LA1 reactors at day 341 of the experiment.

| **Bacteria** | D341 Solid | D347 Solid | D347 Liquid | PP granules | Reactor granules |
| --- | --- | --- | --- | --- | --- |
| PP gr. 0M |  |  |  | 30976 |  |
| PP gr. 6M |  |  |  | 16226 |  |
| Inoculum (12M) |  |  |  | 26071 |  |
| Inf HA | 26050 | 30931 | 17078 |  |  |
| Inf LA | 18555 | 27704 | 14656 |  |  |
| HA1 | 17891 | 40816 | 10066 |  | 22139 |
| HA2 | 23001 | 29858 | 12018 |  |  |
| LA1 | 26136 | 24563 | 23800 |  | 12517 |
| LA2 | 17361 | 34537 | 28865 |  |  |
|  |  |  |  |  |  |
| **Archaea** | D341 Solid | D347 Solid | D347  Liquid | PP granules | Reactor granules |
| PP gr. 0M |  |  |  | 42385 |  |
| PP gr. 6M |  |  |  | 48644 |  |
| Inoculum (12M) |  |  |  | 63835 |  |
| Inf HA | 31317 | 29005 | 55028 |  |  |
| Inf LA | 21764 | 20052 | 40186 |  |  |
| HA1 | 35470 | 39857 | 26827 |  | 36836 |
| HA2 | 36607 | 37466 | 28602 |  |  |
| LA1 | 37887 | 23916 | 36572 |  | 34678 |
| LA2 | 32781 | 45890 | 37538 |  |  |

**Table S2.** OTUs contributing most to the Bray-Curtis dissimilarity between the bacterial communities in the PP granules versus HA1 reactor granules and LA1 reactor granules as identified by SIMPER analysis. Contrib.: contribution; abund.: abundance; p: phylum; c: class; o: order; f: family; g: genus.

| **PP-granules versus HA1 granules** | | | |  |  |
| --- | --- | --- | --- | --- | --- |
| Taxon | Contrib. (%) | Cumulative  (%) | Mean abund. PP  (%) | Mean abund. HA  (%) | Taxonomy |
| OTU 10 | 19.24 | 19.24 | 34.70 | 0.05 | p *Bacteroidetes*; c *Bacteroidia*; o *Bacteroidales* |
| OTU 2 | 8.679 | 27.92 | 0.40 | 16.00 | p *Chloroflexi*; c *Anaerolineae* |
| OTU 13 | 4.638 | 32.55 | 0.001 | 8.34 | k *Bacteria* |
| OTU 27 | 4.445 | 37.00 | 8.31 | 0.32 | p *Bacteroidetes* |
| OTU 6 | 4.252 | 41.25 | 1.67 | 9.31 | p *Aminicenantes*; g *Aminicenantes genera incertae sedis* |
| OTU 2179 | 3.152 | 44.40 | 0 | 5.67 | p *Firmicutes*; c *Clostridia*; o *Clostridiales* |
| OTU 49 | 3.105 | 47.51 | 5.61 | 0.03 | p *Proteobacteria*; c *Deltaproteobacteria*; f *Syntrophorhabdaceae*; g *Syntrophorhabdus* |
| OTU 24 | 2.876 | 50.38 | 0 | 5.17 | p *Firmicutes* |
| **PP granules versus LA1 granules** | | | |  |  |
| Taxon | Contrib. (%) | Cumulative  (%) | Mean abund. PP  (%) | Mean abund. LA  (%) | Taxonomy |
| OTU 10 | 18.68 | 18.68 | 34.70 | 0.02 | p *Bacteroidetes*; c *Bacteroidia*; o *Bacteroidales* |
| OTU 33 | 4.451 | 23.13 | 0.47 | 8.72 | p *Bacteroidetes* |
| OTU 27 | 4.209 | 27.34 | 8.31 | 0.51 | p *Bacteroidetes* |
| OTU 19 | 3.671 | 31.01 | 0.001 | 6.81 | p *Firmicutes*; c *Clostridia*; o *Clostridiales* |
| OTU 45 | 3.39 | 34.40 | 0.16 | 6.45 | p *Synergistetes* |
| OTU 14 | 3.228 | 37.63 | 0.00 | 5.98 | p *Synergistetes*; c *Synergistia*; o *Synergistales*; f *Synergistaceae*; g *Aminobacterium* |
| OTU 49 | 3.018 | 40.65 | 5.61 | 0.02 | p *Proteobacteria*; c *Deltaproteobacteria*; f *Syntrophorhabdaceae*; g *Syntrophorhabdus* |
| OTU 31 | 2.292 | 42.94 | 4.31 | 0.06 | p *Chloroflexi* |
| OTU 24 | 2.198 | 45.14 | 0 | 4.07 | p *Firmicutes* |
| OTU 16 | 2.043 | 47.18 | 0 | 3.79 | p *Cloacimonetes* |
| OTU 144 | 1.823 | 49.00 | 0 | 3.38 | p *Firmicutes*; c *Clostridia* |
| OTU 107 | 1.74 | 50.74 | 3.23 | 0.01 | p *Bacteroidetes* |

**Table S3.** OTUs contributing most to the Bray-Curtis dissimilarity between the archaeal communities in the PP granules, HA1 reactor granules and LA1 reactor granules as identified by SIMPER analysis. OUT 8 and 9 could not be classified even at domain level. Contrib.: contribution; abund.: abundance; o: order; f: family; g: genus.

| PP granules versus HA1 reactor granules | | | | |  | |  |
| --- | --- | --- | --- | --- | --- | --- | --- |
| Taxon | Contrib.  (%) | Cumulative  (%) | Mean abund. PP  (%) | Mean abund. HA1  (%) | | Taxonomy | |
| OTU 10 | 20.04 | 20.04 | 0.002 | 11.90 | | o *Methanomicrobiales*; f *Methanomicrobiaceae*; g *Methanoculleus* | |
| OTU 2 | 17.2 | 37.24 | 40.50 | 30.40 | | o *Methanosarcinales*; f *Methanosaetaceae*; g *Methanosaeta* | |
| OTU 8 | 7.96 | 45.2 | 18.20 | 16.00 | | Unclassified on domain level | |
| OTU 16 | 6.01 | 51.21 | 4.72 | 1.53 | | o *Methanomicrobiales*; f *Methanoregulaceae*; g *Methanolinea* | |
| PP granules versus LA1 reactor granules | | | | |  | |  |
| Taxon | Contrib.  (%) | Cumulative  (%) | Mean abund. PP  (%) | Mean abund. LA1  (%) | | Taxonomy | |
| OTU 2 | 23.79 | 23.79 | 40.50 | 11.40 | | o *Methanosarcinales*; f *Methanosaetaceae*; g *Methanosaeta* | |
| OTU 1 | 15.8 | 39.58 | 0.003 | 19.30 | | o *Methanosarcinales*; f *Methanosarcinaceae* | |
| OTU 3 | 15.71 | 55.3 | 5.74 | 25.00 | | o *Methanobacteriales*; f *Methanobacteriaceae*; g *Methanobacterium* | |

**Figure S1.** Richness and diversity. A) Chao1 for bacterial communities. B) Chao1 for archaeal communities. C) Shannon’s diversity for bacterial and archaeal communities. D) Evenness for bacterial and archaeal communities. The pulp and paper granules (PP gr.) were sampled upon arrival and after 6 and 12 months in storage at 11 °C. The reactor granules were sampled from the HA1 and LA1 reactors at day 341 of the experiment. The liquid fractions were sampled only on day 347.

| A |  |
| --- | --- |
| B |  |
| C |  |
| D |  |
